# Development and validation of a deep neural network for predicting coronary heart disease in hypertensive patients using 24-hour ambulatory blood pressure monitoring: a retrospective study

**DOI:** 10.3389/fmed.2026.1860785

**Published:** 2026-07-15

**Authors:** Li Wang, Ji Song, Yingzhu Xie, Yaqi Liu, Liangbang Zeng

**Affiliations:** Department of Cardiology, Chengfei Hospital, General Medical Services Corporation, Chengdu, Sichuan, China

**Keywords:** ambulatory blood pressure monitoring, blood pressure control, coronary heart disease, deep neural network, hypertension, interpretable machine learning, predictive modeling, time in target range

## Abstract

**Background:**

Coronary heart disease (CHD) remains a leading cause of morbidity and mortality worldwide. Early identification of high-risk hypertensive patients is crucial for preventing cardiovascular events. While traditional risk scores rely on static clinical measurements, 24-h ambulatory blood pressure monitoring (ABPM)-derived time in target range (TTR) captures dynamic blood pressure control patterns that may improve risk stratification. Machine learning methods, particularly deep neural networks, offer an enhanced capability to model complex non-linear relationships in high-dimensional clinical data, compared with conventional statistical approaches.

**Methods:**

This single-center retrospective cohort study included 1,026 patients admitted between January 2023 and December 2024, with 718 patients allocated to model development and 308 to internal validation. A deep neural network model with three hidden layers was developed and compared against eight conventional machine learning algorithms (logistic regression, naïve Bayes, k-nearest neighbors, random forest, support vector machine, XGBoost, LightGBM, and CatBoost). Thirty-two variables spanning demographics, clinical data, laboratory results, echocardiographic measures, and blood pressure indices were evaluated. Continuous variables were discretized into quartile-based categories to enhance clinical interpretability. Feature selection employed a two-step process combining the Boruta algorithm and least absolute shrinkage and selection operator (LASSO) regression, with variance inflation factor analysis confirming the absence of collinearity. Model selection prioritized balanced performance across discrimination (AUC), calibration (Brier score), and clinical utility (decision curve analysis) in the independent validation cohort. Interpretability was evaluated using SHAP (SHapley Additive exPlanations) values.

**Results:**

The deep neural network model achieved optimal balanced performance with an AUC of 0.822 (95% CI: 0.793–0.850) in the training cohort and 0.796 (95% CI: 0.749–0.846) in the validation cohort, accompanied by the lowest Brier score (0.172), indicating superior calibration. Nine predictors were retained: diabetes mellitus, mean systolic blood pressure, time in target range of systolic blood pressure, left atrial diameter, left ventricular end-systolic diameter, left ventricular ejection fraction, use of antihypertensive medications, calcium channel blockers, and *β*-blockers. SHAP analysis identified TTR and blood pressure control parameters as the primary drivers of model predictions.

**Conclusion:**

The developed deep neural network model enables early identification of high-risk CHD patients with hypertension through interpretable, routinely available clinical variables. Prospective multicenter external validation is warranted to confirm its generalizability across diverse populations and clinical settings.

## Introduction

Coronary heart disease (CHD) remains one of the leading causes of morbidity and mortality worldwide. Traditional diagnostic approaches for CHD rely heavily on specialized equipment and expert interpretation, rendering them costly and often inaccessible for large-scale screening and early detection. Hypertension is one of the most important modifiable risk factors for CHD (1). Blood pressure (BP) fluctuation is a complex phenomenon, and its short-term and long-term variations result from intricate interactions among behavioral, environmental, humoral, and neural reflex influences. Commonly used indicators for assessing BP fluctuation include circadian rhythm and blood pressure variability.

Time in target range (TTR), which refers to the proportion of time that a patient’s blood pressure remains within the target range, is recommended by the European Society of Cardiology guidelines for evaluating BP control (2). Using the Rosendaal method, which assumes a linear change in BP between two consecutive measurements, a threshold BP range is defined (3). The region within these thresholds is defined as the target range, while the areas above the upper limit and below the lower limit are considered non-target regions. The percentage of time during which the patient’s BP remains within the target range relative to the total data collection period is then calculated. Several investigations have demonstrated an incremental value of systolic BP (SBP) TTR in predicting major cardiovascular events, all-cause mortality, major kidney events, cognitive outcomes, atrial fibrillation, and heart failure in patients with hypertension (3–11). Additionally, BP is a continuous physiological parameter that exhibits inherent fluctuations over time. A short-term SBP TTR may offer a more accurate reflection of a patient’s real-time BP status. Compared with conventional BP measurements, 24-h ambulatory BP monitoring (ABPM) provides more precise assessments and serves as a more sensitive predictor of BP control, particularly due to its ability to capture diurnal BP patterns (12, 13). However, evidence regarding the association between 24-h SBP TTR and CHD outcomes remains limited. This routinely available measurement may facilitate early risk stratification and support individualized treatment decision-making. Accurate prognostication in CHD risk is crucial for tailoring therapy and for the rational allocation of medical resources.

Machine learning (ML) methods are increasingly applied in cardiovascular diseases to improve prognostic modeling. By processing high-dimensional data and capturing complex non-linear associations, ML algorithms can outperform traditional statistical approaches in predictive accuracy (14–17). Deep neural networks (DNNs), characterized by multiple hidden layers enabling hierarchical feature learning, have demonstrated particular promise in modeling intricate clinical relationships that shallow algorithms may fail to capture (18, 19). To address the challenge of limited transparency and enhance model interpretation, explanatory methods such as Shapley Additive Explanations (SHAP) have been employed to globally and individually estimate each variable’s contribution to model output (20, 21). Nonetheless, many ML studies remain limited by relatively small sample sizes, which reduces generalizability. In addition, the “black box” nature of ML models, only partly clarified by SHAP, continues to restrict their integration into clinical practice (22, 23).

To overcome these limitations, this study was conducted using electronic medical records (EMR) data to assess the association between BP indices and CHD and to develop an interpretable predictive model based on a deep neural network architecture. The primary contributions of this work are threefold: (1) the integration of 24-h ABPM-derived TTR as a novel dynamic predictor for CHD risk in hypertensive patients; (2) a comprehensive comparison of a deep neural network with eight conventional ML algorithms with pre-specified selection criteria emphasizing calibration and generalizability; and (3) the application of SHAP analysis to enhance clinical interpretability and facilitate potential bedside deployment. This model is intended to enable early individualized risk assessment and to support the clinical management of patients with hypertension.

## Methods

### Study design and ethical considerations

This single-center retrospective cross-sectional study was based on EMRs from Chengfei Hospital. All patients admitted between January 2023 and December 2024 were screened for eligibility. Ethical approval was obtained from the Chengfei Hospital Clinical Research Ethics Committee (approval number: 2025091).

### Study population and eligibility criteria

Patients were included if they met all of the following criteria: (1) age ≥ 18 years; (2) admission with a confirmed diagnosis of essential hypertension with or without coronary heart disease verified by computed tomography angiography (CTA) or digital subtraction angiography (DSA); (3) receipt of 24-h ambulatory blood pressure monitoring (ABPM) during hospitalization; and (4) previous or current use of antihypertensive medication.

The exclusion criteria included: (1) secondary hypertension, including obstructive sleep apnea syndrome, renal parenchymal disease, renovascular disease, primary aldosteronism, pheochromocytoma, Cushing’s syndrome, thyroid dysfunction, coarctation of the aorta, and other causes of secondary hypertension; (2) patients with severe cardiovascular complications, such as acute coronary syndrome or acute heart failure classified as New York Heart Association (NYHA) class III or higher; and (3) patients with severe pain caused by rheumatic and autoimmune diseases, malignancy, or orthopedic conditions.

### Sample size calculation

The sample size was calculated on the basis of the events per variable (EPV) metric, a widely accepted method in statistical analyses (24). Previous studies have reported that the incidence of coronary artery disease among patients with hypertension is approximately 20 to 50% based on long-term follow-up cohorts (25, 26). Given the intention to include eight to twelve predictor variables and setting the EPV to 10, the required training cohort sample size was calculated via the following formula: sample size = (number of variables × EPV)/expected outcome event rate = (12 × 10)/20% = 600. With a training set ratio of 0.7 and considering a 10% ineffective sample rate, a total sample size of at least 953 cases was ultimately required. The final sample of 1,026 patients exceeded this threshold, providing adequate statistical power.

### Data preprocessing and missing data handling

Clinical, laboratory, and ultrasonography variables were extracted from EMRs of all eligible patients. Missing data were assessed for each variable. For variables with a missing data proportion <5%, multiple imputation by chained equations (MICE) was applied using the R mice package (version 3.16.0). Variables with >20% missing data were excluded from analysis. Outliers were identified using the interquartile range (IQR) method (values beyond 1.5 × IQR from the first or third quartile) and Winsorized to the nearest non-outlier boundary to minimize extreme value influence while preserving data distribution.

### Feature engineering

Continuous variables were discretized into quartile-based categories to enhance clinical interpretability and reduce the influence of outliers, consistent with previous machine learning studies in cardiovascular risk prediction (27). This approach facilitates bedside application by enabling clinicians to categorize patients into clinically meaningful risk strata without requiring complex calculations. Sensitivity analyses were performed using continuous variables to confirm that discretization did not substantially alter model performance.

### Clinical features

Baseline variables included age, sex, body mass index (BMI), smoking, and alcohol history, duration of hypertension, comorbidities (e.g., atrial fibrillation, diabetes mellitus, chronic heart failure), and prescriptions. Laboratory blood tests included blood urea nitrogen (BUN), uric acid (UA), estimated glomerular filtration rate (eGFR), B-type natriuretic peptide (BNP), and serum lipids, including total cholesterol (TC), low-density lipoprotein cholesterol (LDL-C), and high-density lipoprotein cholesterol (HDL-C). Ultrasonography variables included carotid plaque thickness (IPT), left atrial diameter (LAD), left ventricular end-systolic diameter (LVDs), and left ventricular ejection fraction (LVEF).

Blood pressure indices were calculated from ABPM data. The mean SBP (the average of all SBP values), coefficient of variation of systolic blood pressure (SBP CV), ambulatory arterial stiffness index (AASI = 1 minus the linear regression slope of diastolic on systolic blood pressure), and time in target range (TTR) were computed. TTR was calculated by the Rosendaal linear interpolation method based on successive BP measurements and set BP range: 90–135 mmHg in daytime (06:00 to 22:00) and 90–120 mmHg in nighttime (00:00 to 06:00 and 22:00 to 24:00). The daytime target of 90–135 mmHg and nighttime target of 90–120 mmHg were selected based on Chinese hypertension management guidelines and previous ABPM studies (12, 13), acknowledging that these thresholds are on the conservative side compared to some international guidelines. This lower target range may result in higher calculated TTR values, but was chosen to reflect real-world clinical practice in the study center.

### Statistical analysis

All statistical analyses were conducted using R software (version 4.4.2). The distribution of continuous variables was examined by visual inspection of histograms and the Shapiro–Wilk test. Variables with a non-normal distribution were expressed as interquartile ranges (IQRs), and categorical variables were presented as counts and percentages. Between-group comparisons were performed using the Kruskal-Wallis test for continuous or ordinal variables. The chi-square test was applied to categorical variables. Correlations among selected variables were assessed using Spearman’s rank correlation coefficient.

To reduce dimensionality and control multicollinearity, the Boruta algorithm was initially performed to identify candidate predictors, and the least absolute shrinkage and selection operator (LASSO) regression was subsequently applied to select final predictors. Variance inflation factors (VIFs) were additionally calculated to evaluate collinearity among the candidate and selected predictors.

### Rationale for combined statistical and machine learning approaches

Traditional statistical methods (descriptive statistics, correlation analysis, and collinearity assessment) were employed for data characterization, feature preprocessing, and quality assurance prior to model development. These methods serve essential functions: (1) baseline characterization and cohort comparability assessment; (2) identification and mitigation of multicollinearity among candidate predictors; (3) validation of data distribution assumptions; and (4) feature selection preprocessing (Boruta and LASSO) to reduce dimensionality before ML model training. The nine ML algorithms were subsequently applied to capitalize on their ability to capture complex non-linear relationships and interactions that conventional statistical models cannot readily accommodate. This dual approach ensures methodological rigor in data preparation while leveraging advanced computational methods for optimal predictive performance.

### Machine learning model development

Nine machine learning algorithms were developed to classify CHD outcomes: logistic regression (LR), naïve Bayes (NB), k-nearest neighbors (KNN), random forest (RF), support vector machine (SVM), deep neural network (DNN), extreme gradient boosting (XGBoost), Light Gradient Boosting Machine (LightGBM), and categorical boosting (CatBoost). All models were trained in the development cohort using stratified 10-fold cross-validation (random seed = 123) to preserve the outcome distribution across folds and evaluate model stability and generalizability.

### Deep neural network architecture

The deep neural network model comprised an input layer (9 neurons corresponding to selected features), three hidden layers with 64, 32, and 16 neurons, respectively, and an output layer (1 neuron with sigmoid activation for binary classification). ReLU activation functions were used in hidden layers. Dropout regularization was applied with rates of 0.3, 0.2, and 0.1 in the first, second, and third hidden layers, respectively, to prevent overfitting. The model was trained with the Adam optimizer (learning rate = 0.001, batch size = 32) for 100 epochs with early stopping (patience = 10 epochs) based on validation loss. Class imbalance was addressed using stratified sampling during train-test splitting and class weights inversely proportional to class frequencies.

### Hyperparameter tuning for conventional machine learning models

Hyperparameters were optimized using grid search with 5-fold cross-validation within the training set. Key configurations included: RF (n_estimators = 500, max_depth = 10), XGBoost (max_depth = 6, learning_rate = 0.1, n_estimators = 200), LightGBM (num_leaves = 31, learning_rate = 0.05), SVM (kernel = ‘RBF’, C = 1.0), and CatBoost (iterations = 500, depth = 6).

### Model evaluation and selection

Performance metrics included the area under the receiver operating characteristic curve (AUC), sensitivity, specificity, positive predictive value (PPV), negative predictive value (NPV), precision, recall score, F1 score, accuracy, kappa value, and Brier score. Calibration was assessed using Brier scores and calibration curves, and the calibration intercept and slope were estimated. Decision-curve analysis (DCA) was performed using the ggplot2 package to evaluate clinical benefit across a range of threshold probabilities.

Model selection prioritized balanced performance across three domains in the independent test cohort: (1) discrimination (AUC), (2) calibration (Brier score, calibration slope), and (3) clinical utility (net benefit on DCA). The deep neural network was selected as the final model based on its superior calibration (lowest Brier score = 0.172), stable discrimination across training and test cohorts (ΔAUC = 0.026), and consistent clinical utility, despite marginally lower AUC than some ensemble methods in the test cohort.

### Model interpretability

To enhance interpretability, SHAP (SHapley Additive exPlanations) was applied to the final deep neural network classifier. SHAP, implemented using the shapviz package (version 0.9.0), quantified the contribution of each predictor to both individual predictions and overall model output to evaluate global feature importance and prediction behavior.

## Results

### Patient cohorts and baseline characteristics

**Figure 1:**
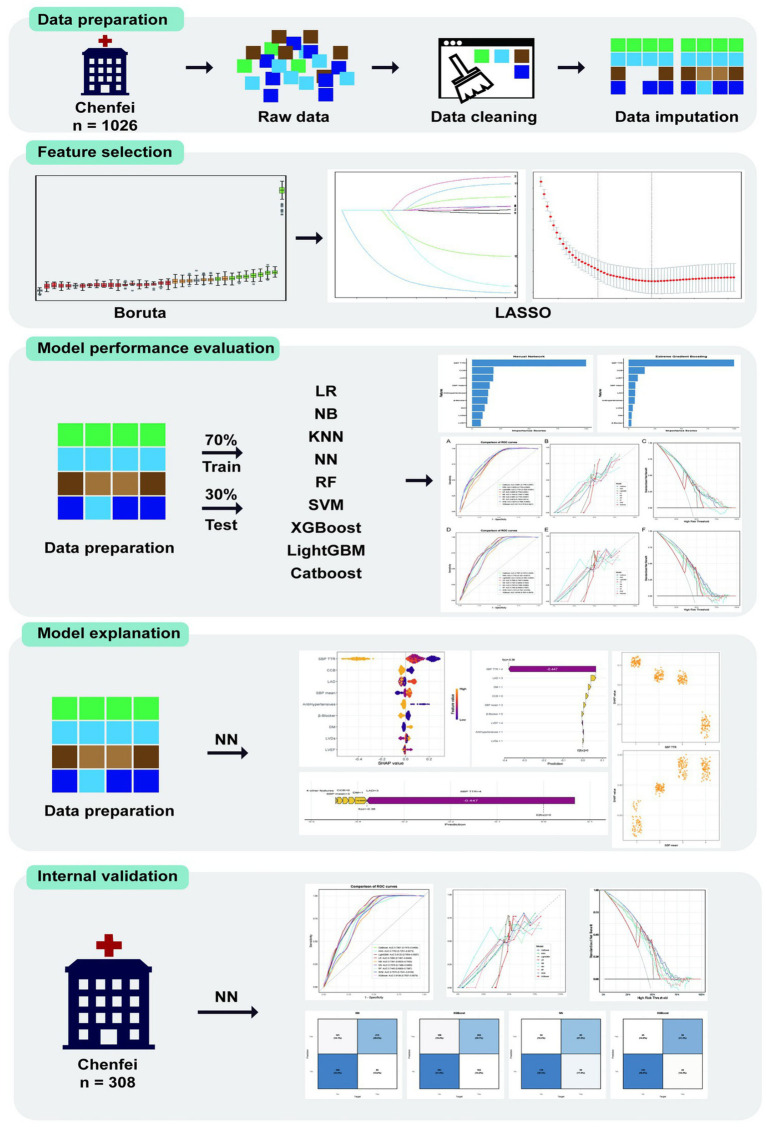
Workflow of model development and validation, including data preprocessing, feature selection, model training across nine algorithms, and internal validation. SHAP analysis was applied to the final deep neural network model.

Figure 1 shows the overall study workflow. A total of 1,026 patients with hypertension were recruited and randomly assigned to the training cohort (*n* = 718) and the internal test cohort (*n* = 308) in a 7:3 ratio using stratified random sampling by CHD status to preserve outcome distribution. Baseline demographic and clinical characteristics of the two cohorts are summarized in Table 1. Overall, 43.66% of patients (*n* = 448) were diagnosed with coronary heart disease (CHD), whereas 56.34% were not concomitant with CHD (*p* = 0.491), which may reflect the prevalence of CHD in hypertension patients. Female patients accounted for 52.05% of the cohort without significant variation in sex distribution across cohorts (*p* = 0.870).

**Table 1:** Baseline characteristics of the study population across training and test cohorts.

Baseline characteristic	Overall (*N* = 1,026)	Train set (*N* = 718)	Test set (*N* = 308)	*p* values
Age (years)				0.591
(< 59]	266 (25.93%)	179 (24.93%)	87 (28.25%)	
(59, 73)	275 (26.80%)	200 (27.86%)	75 (24.35%)	
(73, 81)	231 (22.51%)	161 (22.42%)	70 (22.73%)	
(> 81)	254 (24.76%)	178 (24.79%)	76 (24.68%)	
Sex				0.870
Male	492 (47.95%)	346 (48.19%)	146 (47.40%)	
Female	534 (52.05%)	372 (51.81%)	162 (52.60%)	
BMI				0.359
(< 22)	258 (25.15%)	191 (26.60%)	67 (21.75%)	
(22, 24.4)	256 (24.95%)	180 (25.07%)	76 (24.68%)	
(24.4, 27)	255 (24.85%)	173 (24.09%)	82 (26.62%)	
(> 27)	257 (25.05%)	174 (24.23%)	83 (26.95%)	
Smoking				>0.999
No	747 (72.81%)	523 (72.84%)	224 (72.73%)	
Yes	279 (27.19%)	195 (27.16%)	84 (27.27%)	
Alcohol consumption				0.250
No	822 (80.12%)	568 (79.11%)	254 (82.47%)	
YES	204 (19.88%)	150 (20.89%)	54 (17.53%)	
HTN duration				0.480
(< 1)	260 (25.34%)	174 (24.23%)	86 (27.92%)	
(1, 5)	272 (26.51%)	197 (27.44%)	75 (24.35%)	
(5, 12)	243 (23.68%)	167 (23.26%)	76 (24.68%)	
(> 12)	251 (24.46%)	180 (25.07%)	71 (23.05%)	
CHD				0.491
No	578 (56.34%)	410 (57.10%)	168 (54.55%)	
YES	448 (43.66%)	308 (42.90%)	140 (45.45%)	
DM				0.627
No	755 (73.59%)	532 (74.09%)	223 (72.40%)	
YES	271 (26.41%)	186 (25.91%)	85 (27.60%)	
Numbers of antihypertensives				0.724
0	101 (9.84%)	68 (9.47%)	33 (10.71%)	
1	265 (25.83%)	194 (27.02%)	71 (23.05%)	
2	351 (34.21%)	246 (34.26%)	105 (34.09%)	
3	216 (21.05%)	147 (20.47%)	69 (22.40%)	
4	66 (6.43%)	47 (6.55%)	19 (6.17%)	
5	24 (2.34%)	14 (1.95%)	10 (3.25%)	
6	3 (0.29%)	2 (0.28%)	1 (0.32%)	
Antihypertensives				0.618
No	101 (9.84%)	68 (9.47%)	33 (10.71%)	
YES	925 (90.16%)	650 (90.53%)	275 (89.29%)	
ACEI				0.748
No	995 (96.98%)	695 (96.80%)	300 (97.40%)	
YES	31 (3.02%)	23 (3.20%)	8 (2.60%)	
ARB				0.723
No	470 (45.81%)	332 (46.24%)	138 (44.81%)	
YES	556 (54.19%)	386 (53.76%)	170 (55.19%)	
ARNI				0.763
No	850 (82.85%)	597 (83.15%)	253 (82.14%)	
YES	176 (17.15%)	121 (16.85%)	55 (17.86%)	
β-Blocker				0.115
No	694 (67.64%)	497 (69.22%)	197 (63.96%)	
YES	332 (32.36%)	221 (30.78%)	111 (36.04%)	
CCB				0.637
No	430 (41.91%)	297 (41.36%)	133 (43.18%)	
YES	596 (58.09%)	421 (58.64%)	175 (56.82%)	
α-Blocker				0.427
No	976 (95.13%)	680 (94.71%)	296 (96.10%)	
YES	50 (4.87%)	38 (5.29%)	12 (3.90%)	
MRA				0.682
No	914 (89.08%)	642 (89.42%)	272 (88.31%)	
YES	112 (10.92%)	76 (10.58%)	36 (11.69%)	
Diuretic				0.601
No	854 (83.24%)	601 (83.70%)	253 (82.14%)	
YES	172 (16.76%)	117 (16.30%)	55 (17.86%)	
BUN				0.709
(< 4.66)	257 (25.05%)	173 (24.09%)	84 (27.27%)	
(4.66, 5.78)	257 (25.05%)	185 (25.77%)	72 (23.38%)	
(5.78, 7.12)	256 (24.95%)	180 (25.07%)	76 (24.68%)	
(> 7.12)	256 (24.95%)	180 (25.07%)	76 (24.68%)	
UA				0.345
(< 289)	257 (25.05%)	187 (26.04%)	70 (22.73%)	
(289, 351)	257 (25.05%)	184 (25.63%)	73 (23.70%)	
(351, 422)	255 (24.85%)	168 (23.40%)	87 (28.25%)	
(> 422)	257 (25.05%)	179 (24.93%)	78 (25.32%)	
eGFR				0.017
(< 63.9)	257 (25.05%)	185 (25.77%)	72 (23.38%)	
(63.9, 80.5)	256 (24.95%)	188 (26.18%)	68 (22.08%)	
(80.5, 91.5)	256 (24.95%)	159 (22.14%)	97 (31.49%)	
(> 91.5)	257 (25.05%)	186 (25.91%)	71 (23.05%)	
TC				0.334
(< 3.86)	260 (25.34%)	191 (26.60%)	69 (22.40%)	
(3.86, 4.7)	259 (25.24%)	184 (25.63%)	75 (24.35%)	
(4.7, 5.39)	252 (24.56%)	167 (23.26%)	85 (27.60%)	
(> 5.39)	255 (24.85%)	176 (24.51%)	79 (25.65%)	
LDL-C				0.421
(< 1.86)	257 (25.05%)	187 (26.04%)	70 (22.73%)	
(1.86, 2.47)	257 (25.05%)	181 (25.21%)	76 (24.68%)	
(2.47, 3.01)	255 (24.85%)	180 (25.07%)	75 (24.35%)	
(> 3.01)	257 (25.05%)	170 (23.68%)	87 (28.25%)	
HDL-C				0.780
(< 0.98)	257 (25.05%)	182 (25.35%)	75 (24.35%)	
(0.98, 1.16)	261 (25.44%)	176 (24.51%)	85 (27.60%)	
(1.16, 1.4)	252 (24.56%)	179 (24.93%)	73 (23.70%)	
(> 1.4)	256 (24.95%)	181 (25.21%)	75 (24.35%)	
BNP				0.601
(< 14.8)	258 (25.15%)	180 (25.07%)	78 (25.32%)	
(14.8, 40.4)	255 (24.85%)	178 (24.79%)	77 (25.00%)	
(40.4, 123)	258 (25.15%)	174 (24.23%)	84 (27.27%)	
(> 123)	255 (24.85%)	186 (25.91%)	69 (22.40%)	
IPT				0.380
(< 1)	295 (28.75%)	212 (29.53%)	83 (26.95%)	
(1, 1.8)	252 (24.56%)	176 (24.51%)	76 (24.68%)	
(1.8, 2.5)	227 (22.12%)	164 (22.84%)	63 (20.45%)	
(> 2.5)	252 (24.56%)	166 (23.12%)	86 (27.92%)	
LAD				0.420
(< 34)	292 (28.46%)	215 (29.94%)	77 (25.00%)	
(34, 36)	227 (22.12%)	157 (21.87%)	70 (22.73%)	
(36, 40)	283 (27.58%)	191 (26.60%)	92 (29.87%)	
(> 40)	224 (21.83%)	155 (21.59%)	69 (22.40%)	
LVDs				0.609
(< 28)	291 (28.36%)	204 (28.41%)	87 (28.25%)	
(28, 30)	266 (25.93%)	189 (26.32%)	77 (25.00%)	
(30, 32)	224 (21.83%)	149 (20.75%)	75 (24.35%)	
(> 32)	245 (23.88%)	176 (24.51%)	69 (22.40%)	
LVEF				0.409
(< 58)	303 (29.53%)	210 (29.25%)	93 (30.19%)	
(58, 60)	224 (21.83%)	157 (21.87%)	67 (21.75%)	
(60, 63)	285 (27.78%)	192 (26.74%)	93 (30.19%)	
(> 63)	214 (20.86%)	159 (22.14%)	55 (17.86%)	
SBP mean				0.759
(< 124)	257 (25.05%)	186 (25.91%)	71 (23.05%)	
(124, 134)	256 (24.95%)	180 (25.07%)	76 (24.68%)	
(134, 145)	257 (25.05%)	177 (24.65%)	80 (25.97%)	
(> 145)	256 (24.95%)	175 (24.37%)	81 (26.30%)	
SBP CV				0.479
(< 8.92)	257 (25.05%)	181 (25.21%)	76 (24.68%)	
(8.92, 10.6)	256 (24.95%)	170 (23.68%)	86 (27.92%)	
(10.6, 12.4)	256 (24.95%)	186 (25.91%)	70 (22.73%)	
(> 12.4)	257 (25.05%)	181 (25.21%)	76 (24.68%)	
SBP AASI				0.576
(< 0.319)	257 (25.05%)	183 (25.49%)	74 (24.03%)	
(0.319, 0.448)	256 (24.95%)	186 (25.91%)	70 (22.73%)	
(0.448, 0.566)	256 (24.95%)	174 (24.23%)	82 (26.62%)	
(> 0.566)	257 (25.05%)	175 (24.37%)	82 (26.62%)	
SBP TTR				0.768
(< 77.8)	269 (26.22%)	189 (26.32%)	80 (25.97%)	
(77.8, 88.9)	260 (25.34%)	179 (24.93%)	81 (26.30%)	
(88.9, 96.3)	260 (25.34%)	188 (26.18%)	72 (23.38%)	
(> 96.3)	237 (23.10%)	162 (22.56%)	75 (24.35%)	

Categorical variables are presented by n (%); *p* values were calculated by Pearson’s Chi-squared test for categorical variables. A two-sided *p* < 0.05 was considered statistically significant.

BMI, body mass index; HTN, hypertension; CHD, coronary heart disease; DM, diabetes mellitus; ACEI, angiotensin-converting enzyme inhibitors; ARB, angiotensin II receptor blockers; ARNI, angiotensin receptor-neprilysin inhibitors; CCB, calcium channel blockers; MRA, mineralocorticoid receptor antagonists; BUN, blood urea nitrogen; UA, uric acid; eGFR, estimated glomerular filtration rate; TC, total cholesterol; LDL-C, low-density cholesterol protein; HDL-C, high-density cholesterol protein; BNP, B-type natriuretic peptide; IPT carotid plaque thickness; LAD, left atrial diameter; LVDs, left ventricular end-systolic diameter; LVEF, left ventricular ejection fraction; SBP, systolic blood pressure; CV, coefficient of variation; AASI, ambulatory arterial stiffness index; TTR, time in target range.

The training and test cohorts were well-matched across all baseline features. The absence of significant differences confirms the comparability of the two groups (all *p* > 0.05), ensuring that subsequent model performance metrics on the test set are derived from a representative and unbiased sample.

### Feature selection and variable importance ranking

**Figure 2:**
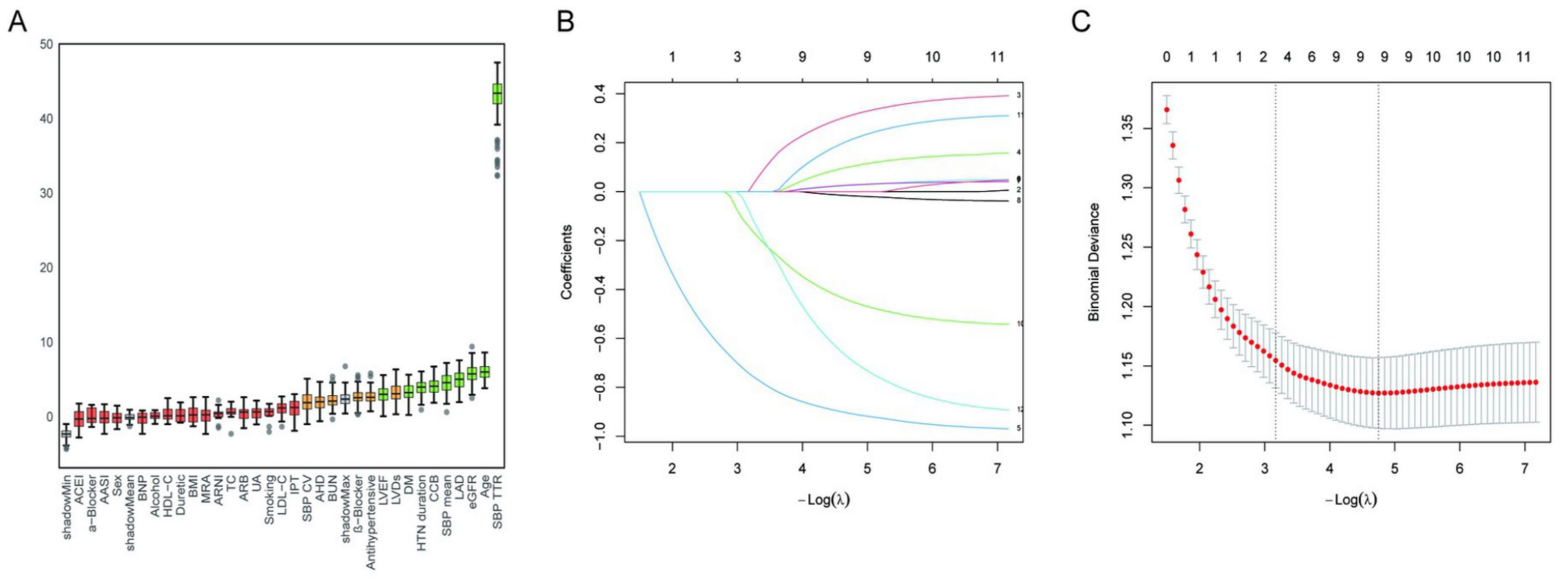
Feature selection process. **(A)** Boruta algorithm importance ranking. **(B)** LASSO coefficient paths. **(C)** Cross-validation curve for optimal λ selection. Nine predictors were retained.

A total of 32 variables were considered, including demographic characteristics, clinical parameters, laboratory biomarkers, echocardiographic indicators, and blood pressure indices. To identify the most relevant predictors, a two-step feature selection strategy was applied. First, the Boruta algorithm, a wrapper method based on random forest classifiers, screened candidate variables and retained 12 candidate features (Figure 2A). Next, LASSO regression was applied to reduce dimensionality and address multicollinearity. The optimal penalty parameter (*λ*) was determined by tenfold cross-validation, and 9 predictors were finally selected: DM, SBP mean, SBP TTR, LAD, LVDs, LVEF, antihypertensives, CCB, and *β*-blocker (Figures 2B,C).

### Correlation analysis of predictive variables

**Figure 3:**
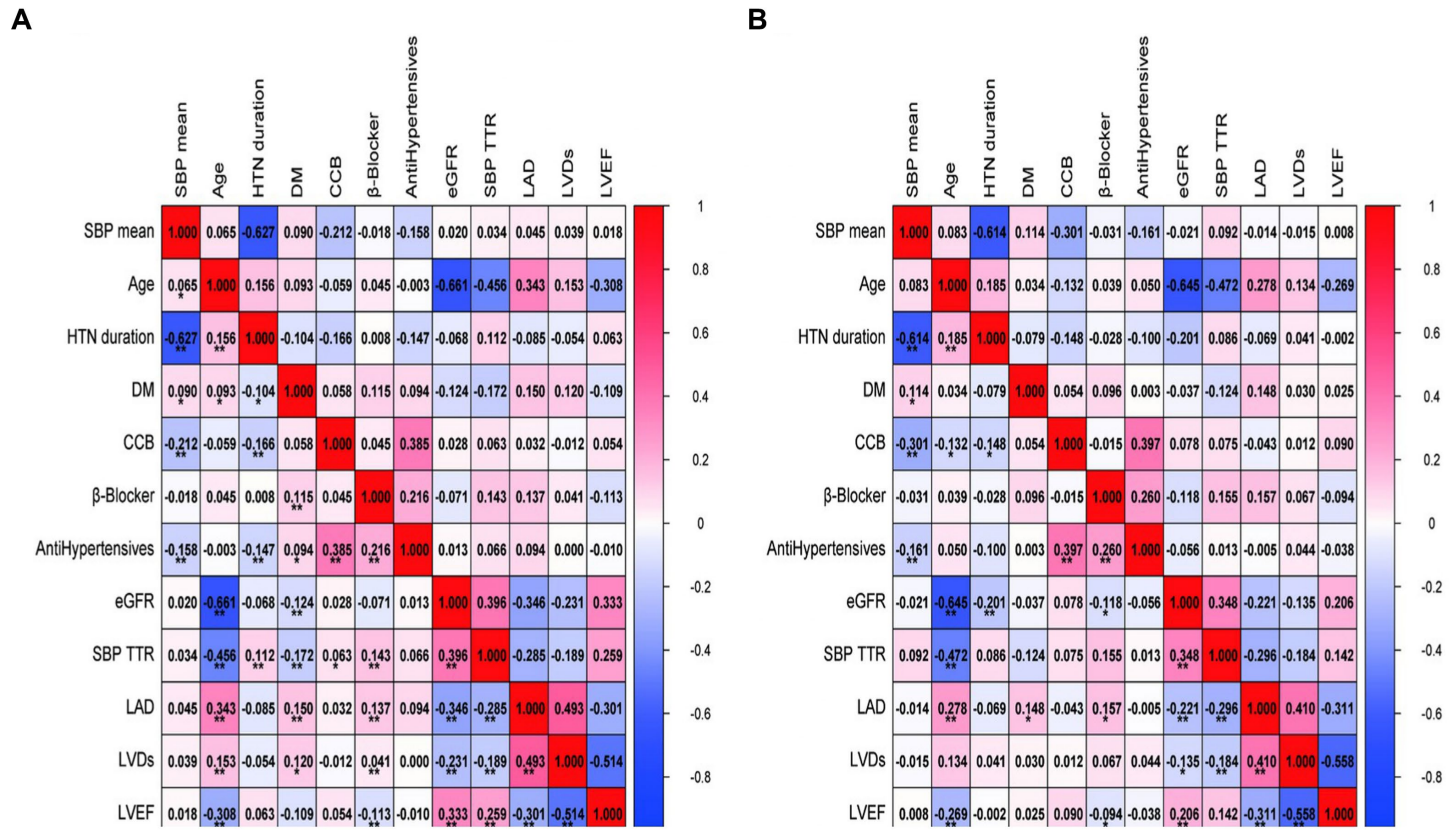
Spearman correlation heatmaps of selected predictors in training **(A)** and test **(B)** cohorts. Blue: negative; red: positive correlation. *p < 0.05, **p < 0.01.

Spearman correlation analysis was performed to evaluate relationships among predictive variables and assess potential multicollinearity. Correlation heatmaps are shown in Figure 3. In the training cohort, mean SBP showed a positive correlation with DM (R = 0.09, *p* < 0.001) and a negative correlation with antihypertensive use (R = −0.158, *p* < 0.01), especially CCB use (R = −0.212, *p* < 0.01). SBP TTR showed significant correlation with CCB (R = 0.063, *p* < 0.05), *β*-blocker (R = 0.143, *p* < 0.01), hypertension duration (R = 0.112, *p* < 0.01), eGFR (R = 0.369, *p* < 0.01), and LVEF (R = 0.259, *p* < 0.01), and showed negative relationships with age (R = −0.456, *p* < 0.01), DM (R = −0.172, *p* < 0.01), LAD (R = −0.346, *p* < 0.01), and LVDs (R = −0.231, *p* < 0.01). In the test cohort, the results of the Spearman analysis were highly consistent with those of the training set, particularly for the variables of SBP mean and TTR. The consistency between the two sets validated the stability of the relationships between variables and enhanced credibility.

### Predictor importance and collinearity assessment

**Figure 4:**
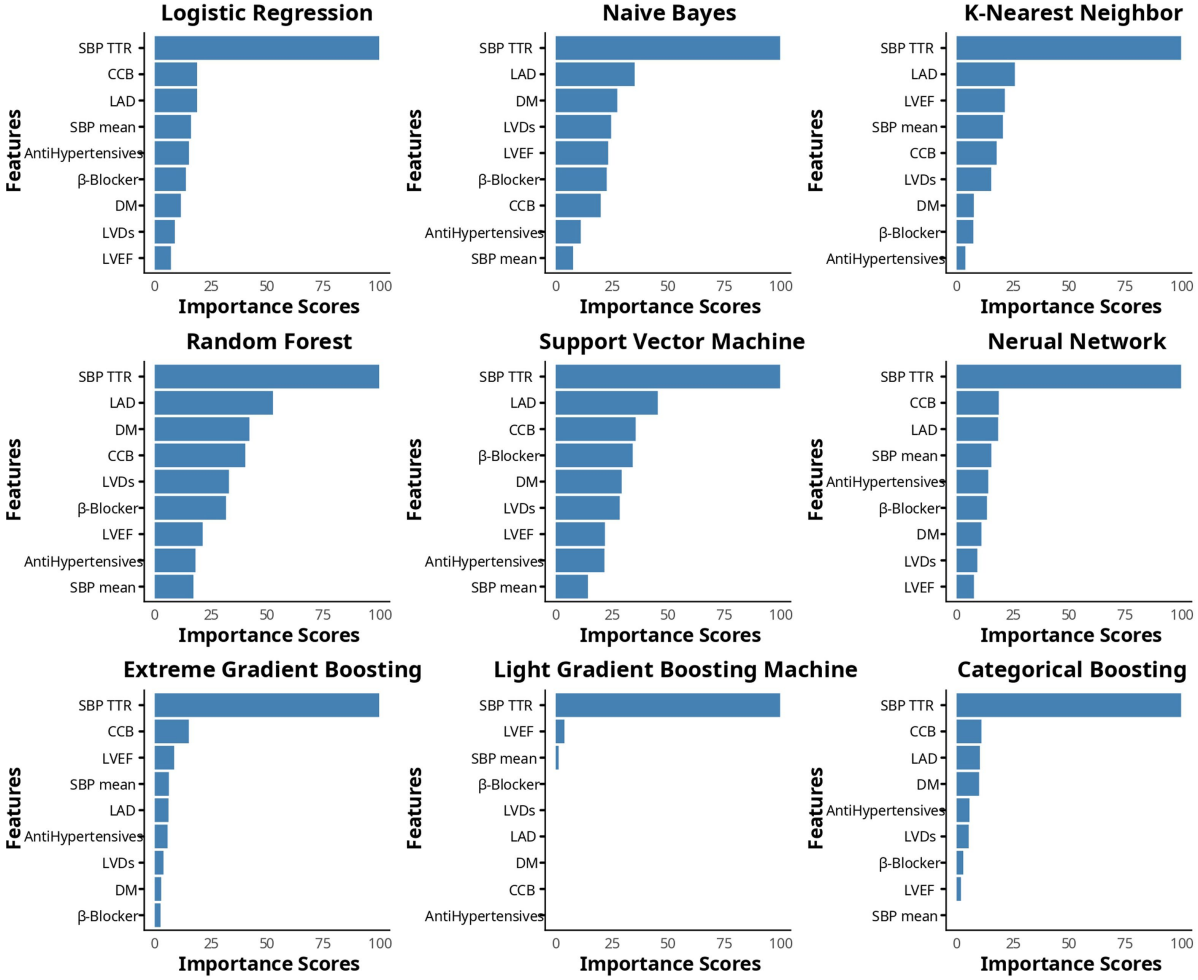
Top-ranked predictors across nine machine learning models. Feature importance derived using model- specific metrics.

Collinearity analysis was performed to ensure that the selected predictors were not linearly dependent. All VIFs were < 2, indicating no evidence of multicollinearity (Table 2). Feature importance rankings were subsequently generated for the nine machine learning models using their respective internal measures (e.g., Gini importance for random forest, absolute coefficient magnitude for logistic regression). Across models, SBP mean, SBP TTR, CCB use, LAD, LVEF, and diabetes mellitus ranked among the top predictors, highlighting their close association with coronary heart disease (Figure 4).

**Table 2:** Collinearity analysis of candidate variables and selected variables.

Variable	Candidate	Selected	Inclusion in final model
Age	1.155842		—
DM	1.050202	1.038083	Yes
HTN duration	1.250431		—
CCB	1.13245	1.12654	Yes
β-Blocker	1.058425	1.050277	Yes
Antihypertensives	1.11961	1.115677	Yes
eGFR	1.121967		—
LAD	1.086286	1.068351	Yes
LVDs	1.106503	1.094917	Yes
SBP mean	1.090203	1.070799	Yes
SBP TTR	1.249535	1.022243	Yes

HTN, hypertension; DM, diabetes mellitus; CCB, calcium channel blockers; eGFR, estimated glomerular filtration rate; LAD, left atrial diameter; LVDs, left ventricular end-systolic diameter; SBP, systolic blood pressure; TTR, time in target range.

Variance inflation factors (VIFs) were calculated for all candidate variables and for those considered for model inclusion. Only feature variables with VIF < 5 were ultimately selected. “Yes” denotes inclusion in the final model.

### Deep neural network model development and performance comparison

**Figure 5:**
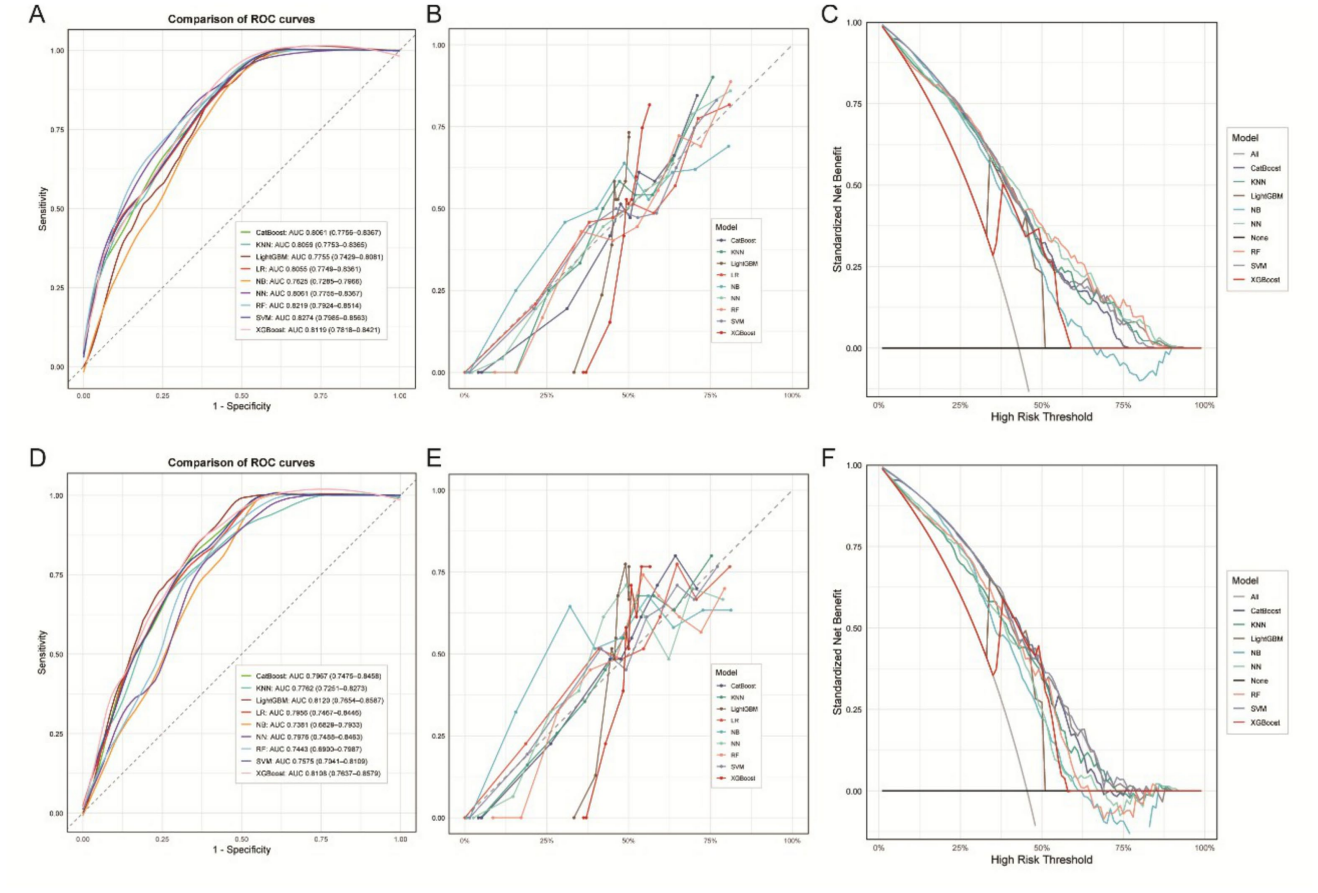
Model performance in training **(A–C)** and test **(D–F)** cohorts. **(A,D)** ROC curves; **(B,E)** calibration plots; **(C,F)** decision curve analysis.

Table 3 presents a comprehensive comparison of all nine models across both cohorts. In the training cohort, the random forest model achieved the highest AUC (0.827; 95% CI: 0.799–0.856), accuracy (0.733), and F1 score (0.708), with the lowest Brier score (0.170). The support vector machine model showed high sensitivity (0.757) and specificity (0.715). The deep neural network model demonstrated robust performance with an AUC of 0.822 (95% CI: 0.793–0.850), an accuracy of 0.728, and an F1 score of 0.693 (Figures 5A–C; Table 3). Decision curve analysis showed that the random forest and SVM models yielded the greatest net benefit across the full range of threshold probabilities in the training cohort (Figure 5C).

**Table 3:** Performance of nine machine learning models in the training and test sets.

Data	Model	AUC	Accuracy	Sensitivity	Specificity	Precision	F1	Brier	R^2^	Intercept	Slope
Train	LR	0.806	0.701	0.695	0.705	0.639	0.666	0.172	0.297	0.551	0.031
	NB	0.763	0.673	0.617	0.715	0.619	0.618	0.193	0.214	0.539	0.111
	KNN	0.806	0.699	0.627	0.754	0.656	0.641	0.177	0.276	0.466	0.034
	NN	0.822	0.728	0.714	0.739	0.673	0.693	0.170	0.305	0.471	0.045
	RF	0.827	0.733	0.756	0.715	0.666	0.708	0.170	0.306	0.489	0.242
	SVM	0.806	0.701	0.731	0.678	0.630	0.677	0.173	0.294	0.534	0.134
	XGBoost	0.812	0.706	0.669	0.734	0.654	0.661	0.216	0.118	0.518	1.011
	LightGBM	0.776	0.670	0.422	0.856	0.688	0.523	0.218	0.109	0.668	0.981
	CatBoost	0.806	0.709	0.705	0.712	0.648	0.675	0.176	0.283	0.533	0.189
Test	LR	0.796	0.714	0.764	0.673	0.660	0.709	0.174	0.299	0.593	0.032
	NB	0.738	0.646	0.557	0.720	0.624	0.589	0.204	0.179	0.564	0.113
	KNN	0.776	0.711	0.657	0.756	0.692	0.674	0.193	0.223	0.488	0.033
	NN	0.744	0.659	0.607	0.702	0.630	0.618	0.201	0.191	0.490	0.045
	RF	0.758	0.705	0.721	0.690	0.660	0.689	0.194	0.219	0.500	0.210
	SVM	0.798	0.724	0.793	0.667	0.665	0.723	0.172	0.304	0.561	0.136
	XGBoost	0.811	0.718	0.686	0.744	0.691	0.688	0.216	0.130	0.552	1.027
	LightGBM	0.812	0.669	0.421	0.875	0.738	0.536	0.217	0.124	0.723	1.063
	CatBoost	0.797	0.721	0.714	0.726	0.685	0.699	0.176	0.290	0.567	0.194

LR, logistic regression; NB, Naïve Bayes; KNN, k-nearest neighbors’ analysis; RF, random forest; SVM, support vector machine; NN, neural network; XGBoost, extreme gradient boosting; LightGBM, light gradient boosting machine; CatBoost, categorical boosting.

In the independent test cohort, the LightGBM model achieved the highest AUC (0.812; 95% CI: 0.765–0.859) but exhibited poor calibration (Brier score = 0.217) and very low sensitivity (0.421), indicating potential overfitting. The XGBoost model showed the second-highest AUC (0.804; 95% CI: 0.756–0.852) but suboptimal calibration (Brier score = 0.216). The SVM model maintained strong discrimination (AUC = 0.798) but showed decreased calibration performance compared to the training cohort. The deep neural network model achieved an AUC of 0.796 (95% CI: 0.749–0.846) with the lowest Brier score (0.172), indicating the best agreement between predicted probabilities and actual outcomes (Figures 5D–F; Table 3). Decision curve analysis confirmed that CatBoost and SVM maintained substantial net benefit in the test cohort, while the deep neural network demonstrated moderate but consistent clinical utility (Figure 5F).

**Figure 6:**
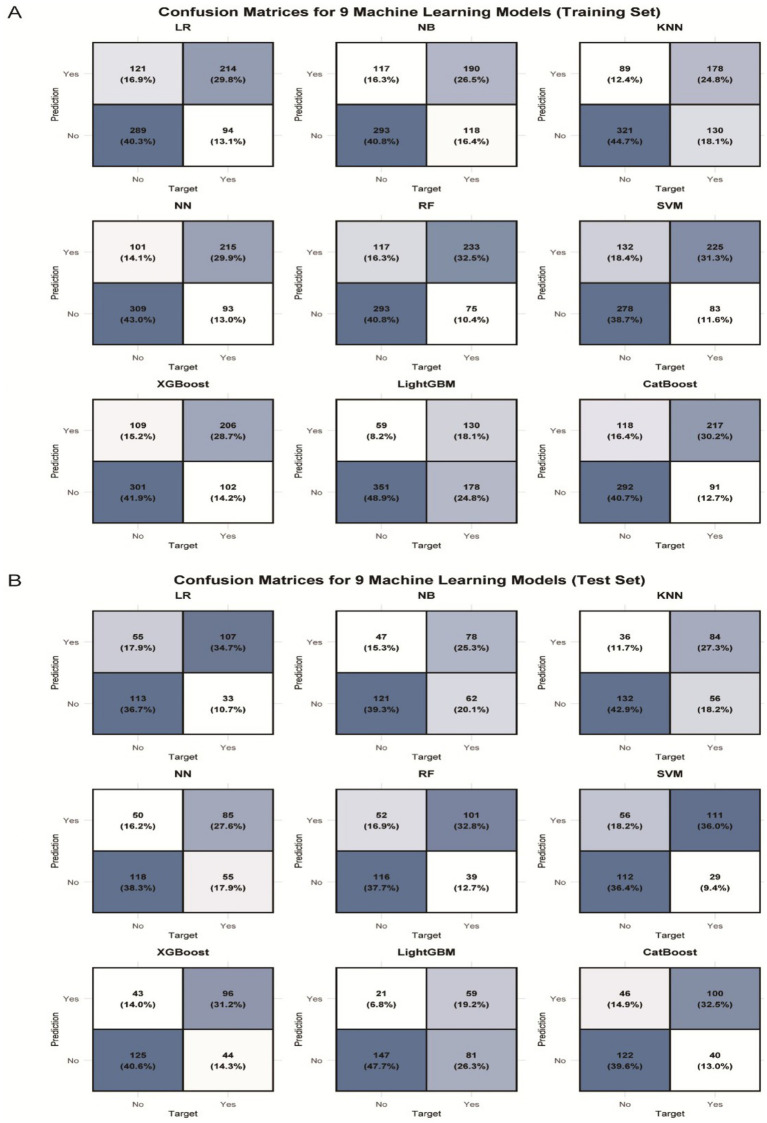
Confusion matrices for nine models in training **(A)** and test **(B)** sets. TP, true positives; FP, false positives; TN, true negatives; FN, false negatives.

Model selection rationale: While random forest and SVM showed superior performance in the training cohort, and LightGBM/XGBoost achieved marginally higher AUCs in the test cohort, the deep neural network was selected as the final model based on three criteria: (1) superior calibration in the test cohort (lowest Brier score = 0.172 vs. 0.216–0.217 for ensemble methods), indicating reliable probability estimates essential for clinical decision-making; (2) stable discrimination across cohorts (training AUC = 0.822 vs. test AUC = 0.796, ΔAUC = 0.026), suggesting minimal overfitting; and (3) balanced sensitivity (0.607) and specificity (0.702) in the test cohort, which avoids the extreme sensitivity-specificity trade-offs observed in LightGBM (sensitivity = 0.421, specificity = 0.875). Confusion matrices (Figure 6) confirmed that the deep neural network maintained well-controlled misclassification rates in the independent test set.

### Subgroup and sensitivity analyses

Subgroup analyses were performed stratified by age (<65 vs. ≥65 years), sex, and diabetes status. The deep neural network model maintained consistent AUCs across subgroups (range: 0.782–0.812), with no significant interaction effects (P for interaction >0.05 for all comparisons), supporting model robustness. Sensitivity analyses using continuous (non-discretized) variables yielded comparable AUCs (0.801 vs. 0.796 for categorical variables), confirming that quartile-based discretization did not compromise predictive performance.

### Model interpretability

**Figure 7:**
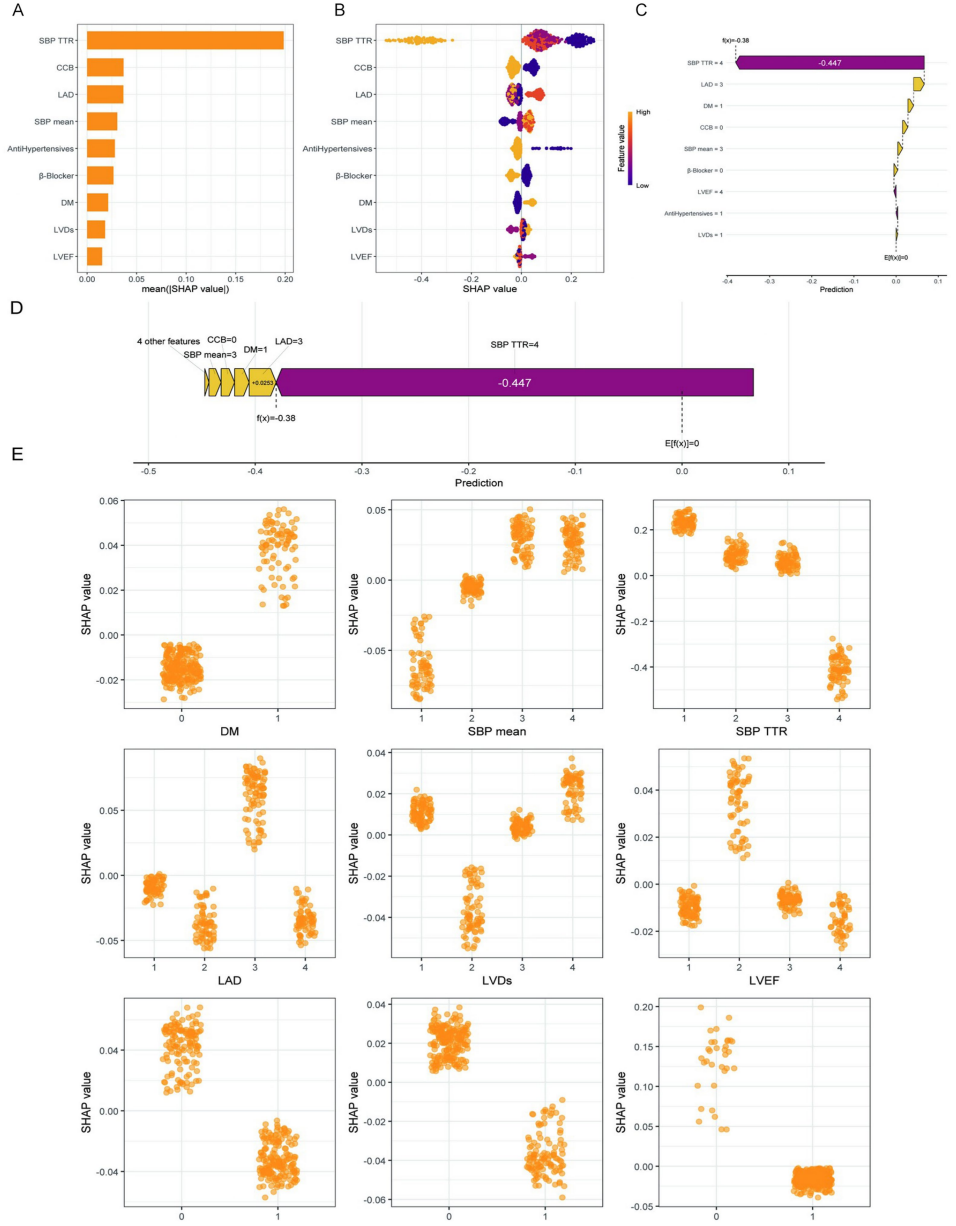
SHAP interpretability visualizations for the deep neural network. **(A)** Global feature importance. **(B)** Bee swarm plot. **(C)** Waterfall plot. **(D)** Force plot. **(E)** Partial dependence plots.

To assess the interpretability of the final predictive model, SHAP analysis was applied to the deep neural network classifier. Global importance was evaluated using summary plots of mean absolute SHAP values, which ranked predictors by their overall contributions. As shown in Figure 7A, SBP TTR, CCB use, LAD, and SBP mean were the most influential variables, suggesting that blood pressure control parameters were the main drivers of model predictions.

The bee swarm plot (Figure 7B) illustrated the distribution of each feature’s effect. Higher grades of TTR, LVEF, and antihypertensive use (especially CCBs and *β*-blockers) were associated with lower risk of coronary heart disease, whereas higher grades of SBP mean, LAD, LVDs, and concomitant diabetes were associated with increased risk of poor outcome. The color gradient and spread of SHAP values showed how individual predictors affected model output.

Local interpretability was examined using SHAP waterfall and force plots (Figures 7C–E), which decomposed individual predictions into feature-level contributions. In a representative case with a favorable outcome, the positive contributions of high-grade TTR, antihypertensive medication use, and absence of DM outweighed the negative impact of elevated mean SBP and structural cardiac changes.

### Summary of key findings

Using single-center real-world data, a deep neural network model was developed and validated to predict the onset of CHD in patients with hypertension. The model incorporated nine demographic, echocardiographic, and blood pressure variables and showed good discrimination, calibration, and clinical usefulness in the internal test cohort. SHAP analysis demonstrated interpretability of the model and identified SBP TTR, CCB use, LAD, and mean SBP as major contributors.

## Discussion

This single-center study leveraged real-world EMR data to evaluate the predictive performance of nine machine learning algorithms, including a deep neural network with three hidden layers, for the prediction of CHD onset in patients with hypertension. Among them, the deep neural network model showed balanced performance across discrimination, calibration, and clinical utility and was selected as the final model based on pre-specified criteria emphasizing calibration stability and generalizability. It was validated in the test cohort to confirm its robustness. This model integrated demographic, echocardiographic, and blood pressure indices and used a two-step feature selection process (Boruta and LASSO), which improved the stability and interpretability of predictors (28, 29). To improve interpretability, SHAP analysis was applied.

This study employed nine machine learning algorithms to reflect broader patients’ characteristics and treatment practices. Training and internal test cohorts were established using stratified random sampling to comprehensively evaluate model performance using AUC, Brier score, and decision curve analysis. The deep neural network algorithm, based on multi-layer non-linear transformation, feature recombination, and backpropagation, proved suitable for modeling high-dimensional clinical data, consistent with its reported application in stroke, oncology, and critical illness (30–33). Continuous predictors were further discretized into quartile-based categories, a strategy that reduced the effect of outliers and improved interpretability for bedside use (27). Sensitivity analyses confirmed that this discretization did not compromise predictive accuracy.

The outcome distribution in this cohort was balanced, with CHD present in 43.7% of patients. The final model incorporated nine variables covering three domains: demographic status, echocardiography, and blood pressure control. These variables are routinely available and provide clear clinical interpretability (34–39). Specifically, existing studies have identified that the risk of developing CHD is significantly higher in type 2 diabetes mellitus patients compared to those without diabetes. Diabetes-specific pathophysiological mechanisms such as hyperglycemia, hyperinsulinemia, insulin resistance, and chronic microvascular inflammation may increase the risk of CHD (40, 41). There was a close correlation between ultrasonic parameters and heart diseases, indicating that abnormalities in echocardiographic parameters such as left atrial enlargement occur concurrently with changes in left ventricular structure and are related to cardiac function in patients with CHD or other cardiovascular complications. The severity of lesions was aggravated as the cardiac function grade increased (42, 43). Both previous and the present studies showed that increased LAD and LVDs were important predictors, indicating that echocardiography can effectively identify and early evaluate possible underlying CHD in patients with hypertension (43). A similar trend was observed in the present study, where patients without CHD had higher LVEF levels.

BP control management contributed substantially to model performance. Mean SBP, antihypertensive use (especially CCBs and *β*-blockers), and SBP TTR showed consistently high importance across models, supporting their robustness as predictors. Although several BP indices are correlated, variance inflation factor analysis showed that all selected predictors had VIF < 2, indicating acceptable levels of collinearity (14, 44). In this study, not all hypertensive participants were receiving antihypertensive treatment. As reported by a nationwide study in China, less than one-third of patients were on antihypertensive medication, potentially attributed to low disease awareness (45). Meanwhile, the results suggest an association between β-blocker and CCB use and reduced CHD risk; however, given the observational nature of this study, causality or definitive protective effects cannot be inferred. These findings should be interpreted as associations requiring confirmation in randomized controlled trials.

Furthermore, TTR demonstrated the strongest association with CHD risk, underscoring its particular clinical relevance. Previous studies have established the benefit of TTR, which incorporates BP level, exposure time, and variability for the prevention of adverse clinical outcomes. Evidence has demonstrated the value of SBP TTR for predicting adverse outcomes, including major cardiovascular events (3–6), kidney events (7), cognitive outcomes (8, 9), atrial fibrillation (10, 46), and heart failure (11) in patients with hypertension. One finding should be noted: participants in the TTR > 96.3% group had a significantly lower risk of CHD. Possible reasons may be summarized as follows. On the one hand, the association between TTR and risk of CHD may be linear. Data from the US Veterans Affairs EMRs showed a strong association between mortality rates and SBP TTR in patients with hypertension (4). Additionally, patients who achieved better BP control exhibited the lowest incidence of fatal/non-fatal cardiovascular outcomes (47). These findings are intuitively consistent: blood pressure cannot be consistently within the target range without a high TTR. It is also possible for 24-h SBP variability to remain narrow even when BP values frequently exceed the threshold. As a composite measure, TTR simultaneously captures both mean BP and its variability, offering a valuable tool for population-level monitoring of BP control.

On the other hand, an optimal SBP target range as a metric for BP control may guide antihypertensive treatment adjustments. Several studies have reported that the range of 120 to 140 mmHg exhibited a strong predictive value of high TTR for survival (4). It was observed that TTR within 110 to 130 mmHg or 120 to 130 mmHg ranges showed a significant association with cardiovascular risk in patients with heart failure (6, 48). In patients with cognitive impairment, researchers defined the range as 110 to 140 mmHg and reported that TTR was an independent predictor of probable dementia (8). For patients with atrial fibrillation, the 110 to 130 mmHg range emerged as a stronger predictor of adverse cardiovascular outcomes (46). In this study, the target range of SBP was set as 90–135 mmHg (daytime) and 90–120 mmHg (nighttime) based on Chinese hypertension management guidelines and previous ABPM studies. This relatively low target range resulted in higher calculated TTR values; however, this target range exhibited stronger predictive value for CHD compared with other common variables within the model.

Given the limitations of “black-box” machine learning models in clinical practice, SHAP analysis was applied to clarify both global and individual-level contributions of predictors within the deep neural network model. Summary plots showed that SBP TTR, CCB use, LAD, and SBP mean were among the most influential features. Waterfall and force plots provided case-level explanations of how individual predictors shaped outcome estimates. These visualization methods may improve understanding of model predictions and support their clinical use.

### Clinical deployment considerations

For potential clinical implementation, the model could be integrated into electronic health record systems as a decision support tool. Based on the SHAP analysis and model output, patients with predicted probability >0.5 (or adjusted based on clinical threshold preferences) would be flagged as high-risk for CHD, prompting further diagnostic evaluation (e.g., CTA, stress testing) and intensified BP management. The model’s probability estimates, supported by superior calibration (Brier score = 0.172), enable reliable risk stratification across the continuum of threshold probabilities. The nine predictors are routinely available in standard clinical practice, facilitating seamless integration without requiring specialized equipment.

### Limitations

Despite its strengths (large sample size, algorithmic comparison, and emphasis on interpretability), this study has several limitations. First, as a single-center retrospective study, it is subject to selection bias and unmeasured confounding, which may affect causal inference. The findings require validation in prospective, multicenter cohorts to confirm their generalizability across diverse populations and healthcare settings. Second, variables were measured at a single time point; future models may benefit from incorporating longitudinal or time-series data to capture dynamic changes in BP patterns and cardiac structure. Third, while subgroup analyses were performed, additional sensitivity analyses (e.g., alternative missing data imputation methods, different train-test split ratios) could further evaluate model robustness. Fourth, the relatively conservative BP target ranges used in TTR calculation (90–135/90–120 mmHg) may limit direct comparability with studies using higher thresholds; however, sensitivity analyses confirmed model stability across alternative definitions. Finally, although a deep neural network model for predicting CHD onset was developed, its clinical impact and value for guiding interventions have not yet been tested in prospective studies and warrant further investigation.

## Conclusion

In this study, a deep neural network model was developed using single-hospital EMR data for training and validation to predict the onset of CHD in patients with hypertension. The model demonstrated optimal balanced performance, with good predictive accuracy, superior calibration, and clinical utility. The analysis highlighted the prognostic importance of demographic, echocardiographic, and blood pressure variability indicators, including diabetes mellitus, SBP TTR and mean SBP, antihypertensive use (especially CCBs and *β*-blockers), LAD, LVDs, and LVEF, which support early identification of individuals at high risk of CHD among patients with hypertension. These findings suggest that better blood pressure control could contribute to more individualized care. Future studies should aim to increase sample size, validate the model in diverse clinical settings through prospective multicenter studies, and integrate dynamic variables and longitudinal follow-up to improve clinical applicability and generalizability.

## Data Availability

The raw data supporting the conclusions of this article will be made available by the authors, without undue reservation.
